# Respiratory health impacts of coal-fired power plant emissions with transboundary environmental complexities

**DOI:** 10.1038/s41598-026-58245-z

**Published:** 2026-06-29

**Authors:** Shyamasree Dasgupta, C. R. Parvathy, Raja Dhar, Sayantan Sarkar, Megha Anand, Akash Sharma, Yingchang You, Shiguo Jia, Rausan Zamir, Mohammad Shoeb, Apostolos Bossios, Joyanto Routh

**Affiliations:** 1 School of Humanities and Social Sciences, Indian Institute of Technology Mandi, Kamand, Himachal Pradesh 175005 India; 2 Department of Pulmonology, Calcutta Medical Research Institute, Kolkata, West Bengal 70027 India; 3 School of Civil and Environmental Engineering, Indian Institute of Technology Mandi, Kamand, Himachal Pradesh 175005 India; 4 Institute for Environmental and Climate Research, Jinan University, Guangzhou, 511443 China; 5 School of Atmospheric Sciences, Sun Yat-sen University, Zhuhai Campus, Guangdong, 519802 China; 6 Department of Chemistry, University of Rajshahi, Rajshahi, 6205 Bangladesh; 7 Department of Chemistry, University of Dhaka, Dhaka, 1000 Bangladesh; 8 Division of Lung and Airway Research, Institute of Environmental Medicine, Karolinska Institutet, Stockholm, Sweden; 9 Karolinska Severe Asthma Center, Department of Respiratory Medicine and Allergy, Karolinska University Hospital, Stockholm, Sweden; 10 Department of Thematic Studies, Linköping University, 581 83 Linköping, Sweden

**Keywords:** Thermal power plant, Particulate matter, Lung health, Transboundary pollution, Socioeconomic conditions, Respiratory tract diseases, Environmental sciences, Environmental social sciences

## Abstract

This interdisciplinary longitudinal study examines the association between PM_2.5_ levels at sites downwind of a coal-fired thermal power plant (TPP) near the India-Bangladesh border and respiratory health in the exposed population, assessed using spirometry and fractional exhaled nitric oxide levels. Pulmonary function tests indicate significantly worse lung health at downwind sites in both India and Bangladesh compared with an upwind location. With averaged forced expiratory volume in one second (FEV_1_) values < 80% of predicted, evidence of preserved ratio impaired spirometry (PRISm), and significantly lower forced expiratory flow between 25% and 75% of vital capacity (FEF_25−75_), the population at the Indian downwind site is clearly at risk of developing obstructive pulmonary and related diseases. Although PM_2.5_ concentrations decline sharply during the monsoon across all sites, this does not translate into a recovery in lung health at the downwind location, consistent with chronic, irreversible effects potentially related to long-term exposure to TPP-associated PM_2.5_. Moreover, poorer socioeconomic conditions and exposure to emissions from biomass-based indoor cooking are associated with exacerbated respiratory effects. Overall, the study advocates transitioning to cleaner fuels for power generation and household use and recommends locating power plants away from densely populated areas to minimize health impacts.

**Supplementary Information:**

The online version contains supplementary material available at 10.1038/s41598-026-58245-z.

## Introduction

Ambient air pollution, one of the most significant global environmental health risk factors, requires sustained attention^1^. Extensive research has shown that exposure to fine and ultrafine particulate matter (PM)^2,3^, ozone (O_3_)^2^, nitrogen oxides (NO_X_)^4^, carbon monoxide (CO)^5^, and other pollutants significantly affect respiratory health, increasing both morbidity and mortality. In particular, studies on ambient PM_2.5_ (particles with aerodynamic diameter ≤ 2.5 μm) exposure indicate that it is responsible globally for ~ 4 million deaths and ~ 118 million disability-adjusted life years (DALYs, a measure of overall disease burden, expressed as the number of years lost due to ill-health, disability, or early death)^6^.

Coal-fired thermal power plants (TPPs) are major sources of PM_2.5_ emissions^7^, impacting air quality and public health^8^, particularly in countries like India, where they account for 70% of the energy generated^9^. These TPPs contribute to poor air quality for ~ 2.3 billion people worldwide, exacerbating asthma, bronchitis, and chronic obstructive pulmonary disease by inducing severe lung inflammation and oxidative stress^10^. Modeling estimates that TPP-associated PM_2.5_ exposure leads to 80,000-115,000 premature deaths, ~ 20 million asthma cases, and an economic burden of $3.2–4.6 billion in India^9,10^. Moreover, PM_2.5_ emitted from TPPs is carried over vast distances by prevailing winds, creating a pollution footprint that extends hundreds of kilometers. Notably, China and India are the top contributors to transboundary air pollution, with India being responsible for significantly higher weighted transboundary pollution than any other country^11,12^. These emissions pose a significant risk to nearby countries, particularly Bangladesh and Nepal, due to their proximity to Indian TPPs, exacerbating air pollution and respiratory health concerns for millions^11^.

The global impact of air pollution is unevenly distributed, with developing economies in Asia and Africa being disproportionately vulnerable in terms of elevated rates of DALYs and mortality, respectively^13–16^. This effect highlights that socioeconomically disadvantaged communities face compounded, cumulative health risks from prolonged exposure to PM due to intersecting vulnerabilities in health, the environment, and access to infrastructure and resources. These communities not only face poorer health outcomes but also experience disproportionately severe impacts from air pollution^17^. Additionally, epidemiological research on respiratory health also reveals gender-based variations in the prevalence, frequency, and clinical consequences of airway diseases^18^. In the global south, including in India, women, who are primarily responsible for cooking, are significantly affected by indoor air pollution due to exposure to smoke from using solid biomass fuels for cooking^19–21^. While contemporary literature shows emerging research related to aerosol distribution and exposure, and its implications for health burdens, there exists a dearth of interdisciplinary research that can aid targeted policy interventions^22^.

Another pressing question is whether emissions from TPPs in India cause respiratory issues in downwind populations and, if so, how prevalent these cases are. Can these effects be distinguished from those arising from other co-located pollution sources, and can we identify the most vulnerable groups? To address these questions, information on community respiratory health is crucial. However, such widespread observations are scarce in India, especially for resource-intensive pulmonary function tests (PFTs), due to public ignorance of symptoms, reluctance to attend health camps, limited healthcare facilities in remote areas, and prohibitive testing costs. Consequently, a large share of the local population exposed to ambient and indoor air pollution goes untested, missing crucial opportunities for early diagnosis of respiratory complications and treatment.

In this interdisciplinary, multinational, longitudinal study, we address this issue by assessing the effects of environmental, socioeconomic, and respiratory health factors among individuals exposed to ambient air pollution downwind of a TPP in India, within a transboundary framework. We use primary longitudinal data and measurements collected across seasons, thereby reaching population groups that are otherwise seldom evaluated for respiratory health. We focus on the following specific questions: (1) Does exposure to ambient air pollution from a coal-fired TPP coexist with deteriorated lung health among nearby populations, and are such trends transboundary? (2) What are the seasonal variations and gender-based disparities in lung health conditions? (3) Can co-located exposures, such as biomass fuel use and smoking, mask the effects of ambient exposure? These questions aim to uncover some of the complexities in assessing and attributing health risks from air pollution near TPPs, particularly in densely populated, underdeveloped, and socioeconomically diverse regions worldwide. Given its interdisciplinary design, the study contributes to the literature by identifying lung health conditions in an otherwise untested population in rural India, linking them to exposure to TPP-related emissions and socioeconomic conditions, and exploring the transboundary nature of the health burden.

## Methodology

A detailed, interdisciplinary framework has been developed to systematically explore the association between the location of coal-fired power plants, air quality and community health, as outlined in our recent work^23^. The current study is a part of this larger work.

### Selection of study sites

**Fig. 1:**
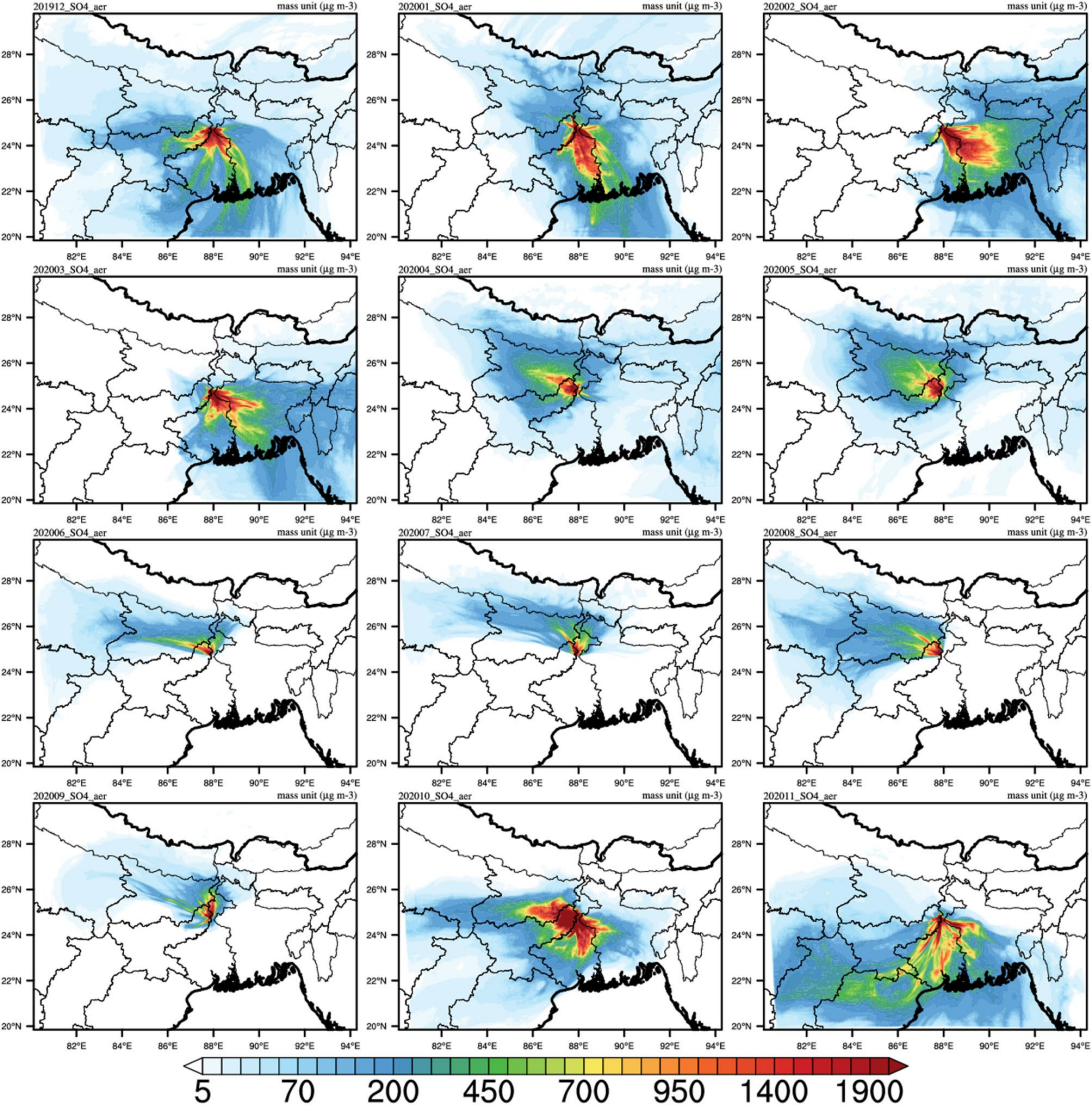
FLEXPART forward simulation of aerosol sulfate (µg m^− 3^; a proxy for PM emissions) from the Farakka thermal power plant in India. Simulations were conducted monthly using WRF-derived meteorological fields from December 2019 to November 2020. Simulations delineate the dominant transport pathways and downwind footprint.

We used the Weather Research and Forecasting (WRF) model to derive meteorological data, followed by the FLEXPART dispersion model to estimate the spatial distribution of the plume from the Farakka TPP in Murshidabad district, West Bengal, India (2100 MW; stack height: 275 m; 24°46’N, 87°53’E) (Fig. 1). The WRF simulation domain consisted of 155 × 122 grid cells centered at 87.5°E, 24.5°N. The horizontal resolution was 9 × 9 km. In the vertical direction, the WRF model was divided into 40 layers from the ground to the model top at 50 hPa. The physical parameterizations adopted in the WRF model included the Purdue Lin scheme for microphysics^24^, the Rapid Radiative Transfer Model for GCMs (RRTMG) for both longwave and shortwave radiation^25^, the Grell 3D for cumulus parameterization^26^, the unified Noah Land Surface Model for land surface processes^27^, the Mellor-Yamada-Janjin (MYJ) boundary layer scheme^28^, and the Building Environment Parameterization (BEP) for the urban surface^29^. The 1°×1° NCEP Final Operational Global Analysis (FNL) dataset was used to provide the initial and boundary conditions of the meteorological fields for the WRF model. The WRF simulation period was from December 2019 to November 2020, and the 9th to the 20th of each month was selected to represent the monthly meteorological conditions. In total, 12 simulation cases were conducted, with each case running for 256 h; the first 16 h were considered spin-up time. The FLEXPART simulation covered the same geographic area as the WRF domain but used a horizontal resolution of 3 × 3 km with 466 × 367 grid cells. The FLEXPART model was configured with 24 vertical layers from the ground up to a height of 2000 m, with a vertical spacing of 25 m below 100 m and a spacing of 100 m above the 100 m level. The release point in the FLEXPART model was defined to represent the TPP.

**Fig. 2:**
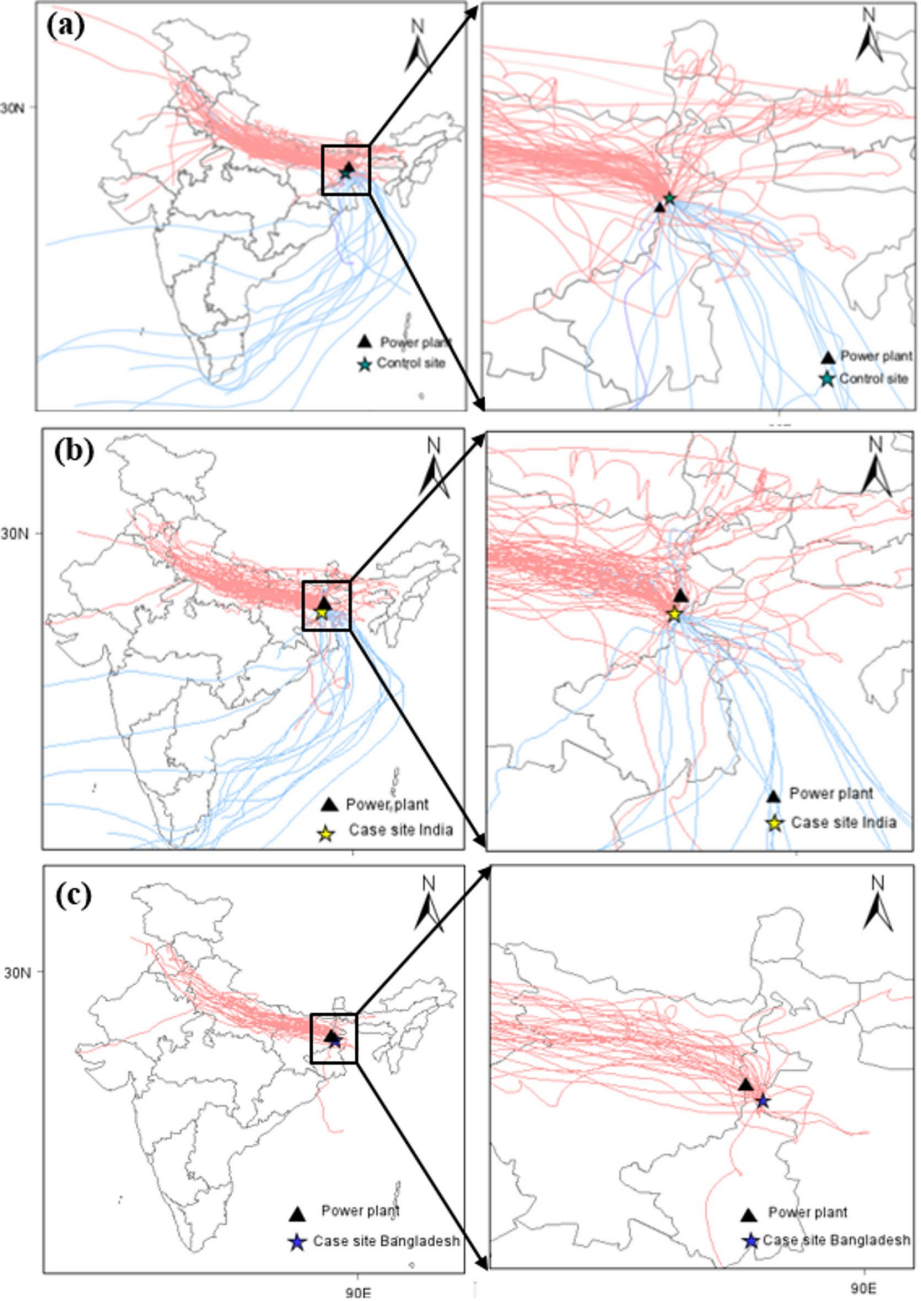
Study area showing the Farakka TPP (black triangle), control site (Malda; green star), case IND (Murshidabad; yellow star), and case BD (Chapai Nawabganj; blue star). HYSPLIT 120-h back trajectories illustrate downwind influence in winters 2021 and 2022 (red) and marine influence in monsoon 2022 (blue).

Positioned ~ 20 km west of the India-Bangladesh border, the super-category coal-fired Farakka TPP, operated by the National Thermal Power Corporation, has operated since 1986 and holds both geographic and economic significance. Its proximity to the densely populated border region makes it a critical energy source and a focal point for potential cross-border air-quality and environmental-health concerns. Based on preliminary model outputs, study sites were strategically identified to examine differences in respiratory health. A location in the upwind direction, largely unaffected by TPP emissions, was chosen as the ‘control site’ and served as a baseline for comparison, providing insights into respiratory health under limited exposure to TPP emissions. Two locations downwind of TPP emissions, one in India and the other in Bangladesh, were referred to as ‘case sites.’ Dispersion results indicated that the TPP plume was transboundary, crossing into neighbouring Bangladesh. Consequently, one control site in Malda district, West Bengal (25°1’N, 88°8’E; ~30 km NE of the TPP) and two case sites, one in Murshidabad district in West Bengal (24°41’N, 87°54’E; ~11 km S-SE of the TPP; case site IND) and one in Chapai Nawabganj, Rajshahi district, Bangladesh (24°35’N, 88°16’E; ~25 km SE of the TPP; case site BD) were selected (Fig. 2). One hundred-twenty-hour air mass backward trajectories, calculated using GDAS meteorological data coupled with the HYSPLIT transport model (https://www.ready.noaa.gov/HYSPLIT.php) (Fig. 2) confirmed that while the control site was unaffected, the two case sites lay downwind of the TPP for the majority of the study period (i.e., approximately 70–80% of trajectory days), and were potentially affected by the emissions plume (winter 2021 and 2022; red lines in Fig. 2). During the 2022 monsoon, trajectories (blue lines in Fig. 2) were predominantly of marine origin from the Bay of Bengal.

### Aerosol sampling and socioeconomic surveys

Aerosol sampling was conducted in winter (December-February) of 2020–2021 and 2021–2022, and during the monsoon (June-August) of 2022 at the control site and case site IND, and during winter 2021–2022 at the case site BD. Samplers were positioned away from high-traffic zones (highways and intersections) and industrial clusters to avoid source-specific bias. We collected 170 aerosol samples during this period at the Indian sites and 34 samples at the Bangladesh site, providing a comprehensive dataset for analysis (season-wise distribution provided in Table 1). Section S1 in the Supplementary Information provides details of the sampling instruments and PM_2.5_ loadings.

We administered household-level socioeconomic surveys, followed by spirometry and FeNO testing on a subset of consenting participants near the samplers to enable an environment–health linkage. The surveys collected information on households’ socioeconomic status, including income, education, and lifestyle factors that could influence respiratory health. One hundred households were surveyed at each location (some were excluded because the respondent left the interview midway), yielding data from more than 400 individuals at each site (Table 1). Households were selected using simple random sampling (SRS) from electoral lists of the village panchayats (administrative units) where the aerosol samplers were located. SRS, as opposed to snowball or purposive sampling, eliminates accessibility-related biases and ensures that individuals who would otherwise be overlooked are included. At a 5% significance level and a 5% margin of error, the sample is representative of up to ~ 30,000 subjects. In Bangladesh, a smaller sample size of 77 individuals was surveyed.

### Pulmonary function tests and respiratory health parameters

We performed spirometry and FeNO on consenting subjects. The measured parameters reflect the subject’s lung physiology, including volume and airflow limitation, if any, and the presence of Type 2 airway inflammation, as indicated by FeNO. These measures are widely accepted for studying the association between air pollution exposure and respiratory health. Table 1 provides the number of acceptable spirometry and FeNO measurements across sites during each round. The spirometry and FeNO records obtained in India over the different sampling events are longitudinal, i.e., tests were conducted repeatedly on the same subjects during different seasons. Due to logistical issues, FeNO could not be measured in the winter 2021 campaign. On some occasions, subjects were unwilling to participate or were unavailable during later rounds, leading to inter-round variation in the number of subjects. All results were screened for test quality, and only valid tests were included in the current analysis (see details in Section S1 of the Supplementary Information).

#### Spirometry measurements

Spirometry, a vital tool for evaluating lung function^30–32^, investigates airway narrowing and distinguishes between obstructive airway disorders, such as Chronic Obstructive Pulmonary Disease (COPD) and asthma, and restrictive diseases, including fibrotic lung disease. Spirometry also aids in tracking disease progression and the severity of respiratory complications. The key measurements provided by spirometry are forced vital capacity (FVC), forced expiratory volume in 1 second (FEV_1_), the FEV_1_/FVC ratio, and the forced expiratory flow between 25% and 75% of vital capacity (FEF_25−75_). Interpretation of the test results considers percentage-predicted values of the parameters, which provide comparative data for an individual subject against reference (predicted) values based on healthy subjects with the same anthropometric parameters (e.g., sex, age, height, and ethnicity, if relevant)^33^. Table S1 in the Supplementary Information summarizes the diagnostic parameters used to assess respiratory health in individuals.

**Table 1:** Seasonal and site-wise distribution of samples and surveys (panel a), with summary statistics for socioeconomic variables and PM_2.5_ at the control site, case IND, and case BD (panel b). Indian sites include winter 2021, monsoon 2022, and winter 2022; the Bangladesh site includes winter 2022. Income in Bangladesh is shown in INR-equivalent terms. Sampling and instrument details are indicated in the Supplementary Information (Section S1).

(a)	Control Site						Case Site IND					Case Site BD					
Sample type	Winter 2021	Monsoon 2022		Winter 2022			Winter 2021	Monsoon 2022			Winter 2022	Winter 2021			Monsoon 2022	Winter 2022	
PM_2.5_ samples collected	34^§^	12^§^		37			39^§^	16^§^			29	-			-	34	
Households surveyed	92	93		95			92	92			92	-			-	23	
Individuals surveyed	416	419		424			459	459			456	-			-	77	
Acceptable spirometry	71	57		45			66	30			49	-			-	69	
Acceptable FeNO	NA	47		38			NA	17			37	**-**			**-**	36	

(b)																	
Variable	Season		Control Site			Case Site IND*			Case Site BD**			p-values for control site v/s case site IND			p-values for control site v/s case site BD		
Average PM_2.5_ concentration (in µg m^−3^)	Winter 2021		139.7 ± 29.7^§^			153.9 ± 45.4^§^						0.06*					
	Monsoon 2022		45.5 ± 11.2^§^			47.4 ± 12.9^§^						0.34					
	Winter 2022		168.5±39.9			185.6±34.8			210±60.3			0.04**			0.00***		
Age (in years)	Winter 2021		40.46 ± 14.86			40.97 ± 15.52						0.84					
	Monsoon 2022		40.49 ± 15.53			44.14 ± 14.11						0.29					
	Winter 2022		43.70 ± 16.6			41.3 ± 14.0			42.1 ± 15.1			0.44			0.58		
Mean per capita income (INR)^	Winter 2021		3787 ± 2302			3085 ± 2179						0.07*					
	Monsoon 2022		5585 ± 9057			3389 ± 2368						0.11					
	Winter 2022		3776 ± 2554			2274± 1471			3506 ± 1285			0.05**			0.88		
Average years of schooling	Winter 2021		8.37 ± 5.5			5.07 ± 4.8						0.00***					
	Monsoon 2022		7.51 ± 5.46			5 ± 4.7						0.05**					
	Winter 2022		9.0 ± 5			3.7 ± 4.3			7.2 ± 5.5			0.00***			0.08*		
% of males^#^ who smoke (self-reported)	Winter 2021		9%			13%						0.3					
	Monsoon 2022		13%			14%						0.9					
	Winter 2022		9%			16%			9%			0.08*			0.99		
% of households using biomass	Winter 2021		63%			89%						0.00***					
	Monsoon 2022		69.40%			80%						0.33					
	Winter 2022		96%			93%			96%			0.65			0.96		

*, **, *** represent statistically significant differences at 10%, 5%, and 1% levels of significance, respectively. ^For Bangladesh, income is given as INR-equivalent; ^#^Only male respondents reported tobacco-smoking; NA: not available. The approximate time interval between two consecutive tests (i.e., between winter 2021 and the monsoon 2022, and between the monsoon 2022 and winter 2022) is six months. ^§^Also reported in Luo, et al.^34^.

#### Fractional exhaled nitric oxide measurements

Elevated FeNO levels indicate inflammation in the airway mucous lining, which can contribute to conditions such as asthma^30^. FeNO levels greater than 50 ppb indicate uncontrolled airway inflammation, whereas those between 25 and 50 ppb suggest inflammation that medication can control. FeNO < 25 ppb suggests the absence of inflammation.

### Statistical analysis

The statistical analysis included: (1) descriptive statistics for socioeconomic indicators, PM_2.5_ levels, spirometry, and FeNO parameters; (2) t-tests comparing lung health across sites and seasons; and (3) kernel density plots for wage loss and productivity (workday) loss. The analyses were conducted using STATA 16.1. See Sect.  1 of the Supplementary Information for more details.

## Results

### PM_2.5_ characteristics at the case and control sites

During winter 2022, the average PM_2.5_ concentrations at case sites IND (185.6 ± 34.8 µg m^− 3^) and BD (210 ± 60.3 µg m^− 3^) were significantly higher (*p* < 0.05) than at the control site (168.5 ± 39.9 µg m^− 3^) when air masses were predominantly north-westerly, and the sites were downwind of the TPP. A similar trend was observed in winter 2021, with average concentrations at the case site IND (153.9 ± 45.4 µg m^−^^3^) higher than at the control (139.7 ± 29.7 µg m^− 3^, with *p* ≤ 0.10)  (see Table 1). During the monsoon, PM_2.5_ concentrations were reduced by factors of 3–4 at both the case (47.4 ± 12.9 µg m^− 3^) and control (45.5 ± 11.2 µg m^− 3^) sites in India, underscoring the importance of precipitation scavenging of aerosols. Overall, a noticeable seasonal footprint of TPP emissions was observed at downwind locations in both India and Bangladesh.

### Socioeconomic characteristics

The demographic and socioeconomic characteristics of the subjects at the case and control sites are presented in Table 1. Mean per-capita incomes are similar at the control site and case BD, but lower at case IND; the difference is moderately significant across all seasons. Mean educational attainment is also significantly higher at the control site than at both case sites in IND and BD. These differences will enable the study to investigate the concept of triple jeopardy further, as, in addition to higher ambient air pollution, the case site IND bears a disproportionate burden in socioeconomic characteristics, such as lower income and education levels. Average ages are comparable across sites and seasons, ranging from 40 to 44 years. The self-reported proportions of smokers (including those who used to smoke but have quit) are also comparable across the locations, with 13% at the case site IND and 9% at both the control site and case site BD. During Winter 2021, biomass use (89%) was significantly higher at the case site IND than at the control site (63%). However, during Monsoon 2022 and Winter 2022, no significant difference was observed in biomass use, which remained in the range of 93–96% across all three sites. Differences across seasons in certain socioeconomic variables are observed because, during the 2022 campaigns, some households and individuals did not consent to spirometry.

### Overall poor respiratory health at the case sites

The average FVC, FEV_1_, FEV_1_/FVC, and FEF_25−75_ across seasons are lower at the case site than at the control site, indicating relatively poor respiratory health (Table 2). In most cases, the average values of spirometry parameters are lowest at case site IND, followed by case site BD (in winter 2022), and are best preserved (i.e., in the best condition) at the control site. The differences are more pronounced when comparing the case site IND with the control site than when comparing the case site BD with the control site. Interestingly, the differences are also substantial between case site IND and case site BD, with the former showing worse respiratory health despite comparable PM_2.5_ concentrations (191 ± 42.2 µg m^−3^ and 198 ± 49.0 µg m^−3^, respectively, in winter 2022). Notably, the proportions of smokers and biomass users are broadly comparable between the control site and the case site IND (other than winter 2022 for smokers and winter 2021 for biomass burning) (Table 1), making it unlikely that these confounders alone explain the non-uniform spirometry outcomes across sites.

**Table 2:** Comparison of spirometry parameters and FeNO across all sites during winter 2021, monsoon 2022, and winter 2022.

	Spirometry parameters/FeNO	Control site (n = 71)	Case site IND (n = 66)	*p*-value for the difference between the control site and the case site IND		
Winter 2021	FVC (%)	82.0 ± 16.4	78.6 ± 17.4			
	FEV_1_(%)	80.2 ± 17.1	74.6 ± 18.1	0.03**		
	FEV_1_/FVC – best trial	82.5 ± 8.1	79.6 ± 10.3	0.03**		
	FEV_1_/FVC (%)	100 ± 9.0	96.8 ± 12.0	0.02**		
	FEF 25–75(%)	74.1 ± 32.8	67 ± 30.0	0.09*		
	FeNO (ppb)	NA	NA	NA		
	**Spirometry parameters/FeNO**	**Control site (n = 57)**	**Case site IND (n = 30)**	**p-value for the difference between the control site and the case site IND**		
Monsoon 2022	FVC (%)	86.3 ± 21.0	76.4 ± 17.4	0.01***		
	FEV_1_(%)	86.1 ± 21.8	72.0 ± 19.4	0.00***		
	FEV_1_/FVC – best trial	84.5 ± 5.7	78.6 ± 8.9	0.00***		
	FEV_1_/FVC (%)	104 ± 9.1	98.4 ± 10.5	0.00***		
	FEF 25–75(%)	73.3 ± 24.0	54.9 ± 24.2	0.00***		
	FeNO (ppb)	21.4 ± 6.3	13.6 ± 11.2	0.00***		
	**Spirometry parameters/FeNO**	**Control site (n = 45)**	**Case site IND (n = 49)**	**p-value for the difference between the control site and the case site IND**	**Case site BD (n = 69)**	**p-value for the difference between case site IND and case site BD**
Winter 2022	FVC (%)	84.1 ± 15.0	77.0 ± 13.4	0.00***	81.6 ± 17.9	0.05**
	FEV_1_(%)	78.3 ± 15.9	71.8 ± 16.3	0.03**	77.9 ± 18.6	0.05**
	FEV_1_/FVC - best trial	79.1 ± 9.6	78.4 ± 9.8		80.1 ± 9.7	
	FEV_1_/FVC (%)	97.9 ± 11.3	97.4 ± 11.3		99.9 ± 11.5	
	FEF 25–75(%)	59.9 ± 24.1	55.2 ± 24.1		63.6 ± 25.7	0.06*
	FeNO (ppb)	21.2 ± 14.3	18.7 ± 15.8		26.3 ± 25.0	0.06*

*^,^**^,^***represent statistically significant differences at 10%, 5%, and 1% levels of significance, respectively. Note 1: differences are not statistically significant between the control site and case site BD for any parameter during winter 2022 and hence are not shown in the table; NA: not available. Note 2: FEV_1_/FVC – best trial refers to the ratio calculated from the single best-effort spirometry trial. i.e., the trial with the highest FVC, expressed as a raw proportion. ‘FEV1/FVC (%)’ refers to the percent-predicted value, i.e., the ratio expressed as a percentage of the expected value for a healthy individual with the same sex, age, height, and ethnicity.

As shown in Table 2 average FEV_1_ values are consistently and significantly lower (*p* < 0.05) at the case site IND (74.6 ± 18.1, 72.0 ± 19.4, and 71.8 ± 16.3 during winter 2021, monsoon 2022, and winter 2022, respectively) compared to the control site (80.2 ± 17.1, 86.1 ± 21.8, and 78.3 ± 15.9 in the same order). FEV_1_ values at the case site IND are lower than those at the control site, yet they remain within the 70–79% range across seasons, suggesting mild obstructive airway disease. A similar trend is observed for FVC values. The average FVC values at the case site IND (78.6 ± 17.4, 76.4 ± 17.4, 77 ± 13.4 during winter 2021, monsoon 2022, and winter 2022, respectively) are lower than those at the control site (82.0 ± 16.4, 86.3 ± 21.0, 84.1 ± 15.0 in the same order), with a statistically significant difference (*p* < 0.01). The results fall within the 70–79% range, indicating potentially reduced lung capacity at the case site. Lower FVC and FEV_1_ profiles at the case site IND, along with preserved FEV_1_/FVC ratios at > 70%, are consistent with the recently developed concept of preserved ratio impaired spirometry (PRISm)^35^. Although not statistically significantly different from the control site (except in monsoon 2022), the lower average FEF_25-75_ at the case site IND (< 65% in monsoon and winter 2022, and 67% in winter 2021) suggests potential small-airway complications, consistent with early COPD. In monsoon 2022, PM_2.5_ levels decline across locations, and the case site IND shows values like those at the control site. However, all spirometry parameters remain significantly lower (*p* ≤ 0.01) at the case site IND, indicating non-uniform seasonal effects on respiratory health.

The average FeNO level is mildly elevated only at the case site BD (26.3 ± 25.0 ppb) in winter 2022. FeNO values are higher at the control site (21.4 ± 6.3 ppb in the monsoon of 2022 and 21.2 ± 14.3 ppb in winter 2022) than at the case site IND (13.6 ± 11.2 ppb in the monsoon of 2022 and 18.7 ± 15.8 ppb in winter 2022); the difference is significant during the monsoon of 2022. This suggests that no definitive conclusions can be drawn from these trends. While FeNO levels reflect eosinophilic airway inflammation, they depend on multiple factors besides PM_2.5_. Emerging evidence suggests that gaseous pollutants, particularly NO_2_ and SO_2_, may also exert a strong eosinophilic/type-2 inflammatory signal in the airways^36–38^. However, values are within the permissible range (< 25) at both sites, indicating no active airway inflammation.

**Fig. 3:**
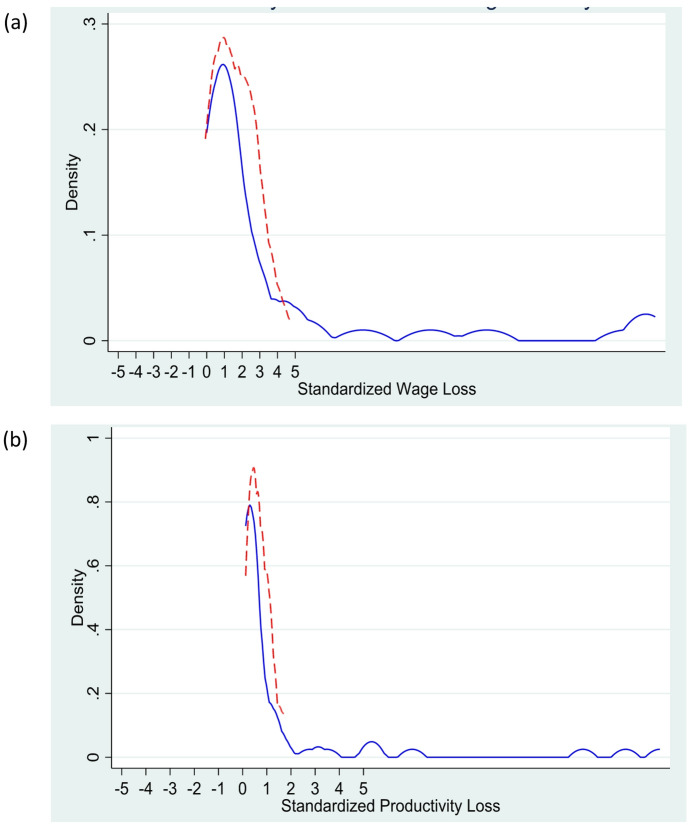
Kernel-density plots of standardized (**a**) wage loss and (**b**) productivity loss at case site IND (red dashed) and control site (blue solid).

Greater impairment of lung health at the case site IND is also associated with a higher probability of wage and productivity losses. As depicted in Fig. 3, except for a few extreme values towards the right tail of the distribution, the kernel density plots of both wage and productivity loss for the case site IND (red broken line) lie above the respective plots for the control site (blue solid line). This suggests that a few individuals at the control site may face very high wage losses. However, considering the larger population, the probability of such losses is unambiguously higher at the case site IND.

The difference in respiratory health between the case sites IND and BD, despite comparable PM_2.5_ concentrations, is intriguing, and confounding factors are likely at play. During winter 2022, spirometry parameters were worse at the case site IND than at the control site, whereas they were comparable between the control site and the case site BD. This pattern is consistent with income and schooling at the control site being significantly higher than at the case site IND but comparable to the case site BD. For example, the case site IND has markedly lower income and educational attainment than the control site (INR 2,274/- vs. INR-equivalent 3,776/- and 3.7 years of schooling vs. 7.2 years, respectively), suggesting that a greater socioeconomic burden may have amplified biological susceptibility to pollution at the case site IND. This association is supported by the fact that in winter 2022, the percentages of households using biomass were comparable across sites, whereas the percentages of smokers differed only weakly significantly. Hence, these confounding factors are unlikely to be the sole contributors to the worsening of respiratory health in the study area. Due to logistical constraints, data from Bangladesh could be collected only for one season, thereby limiting the scope of detailed analysis of confounders across seasons.

### Effect of seasonality

**Fig. 4:**
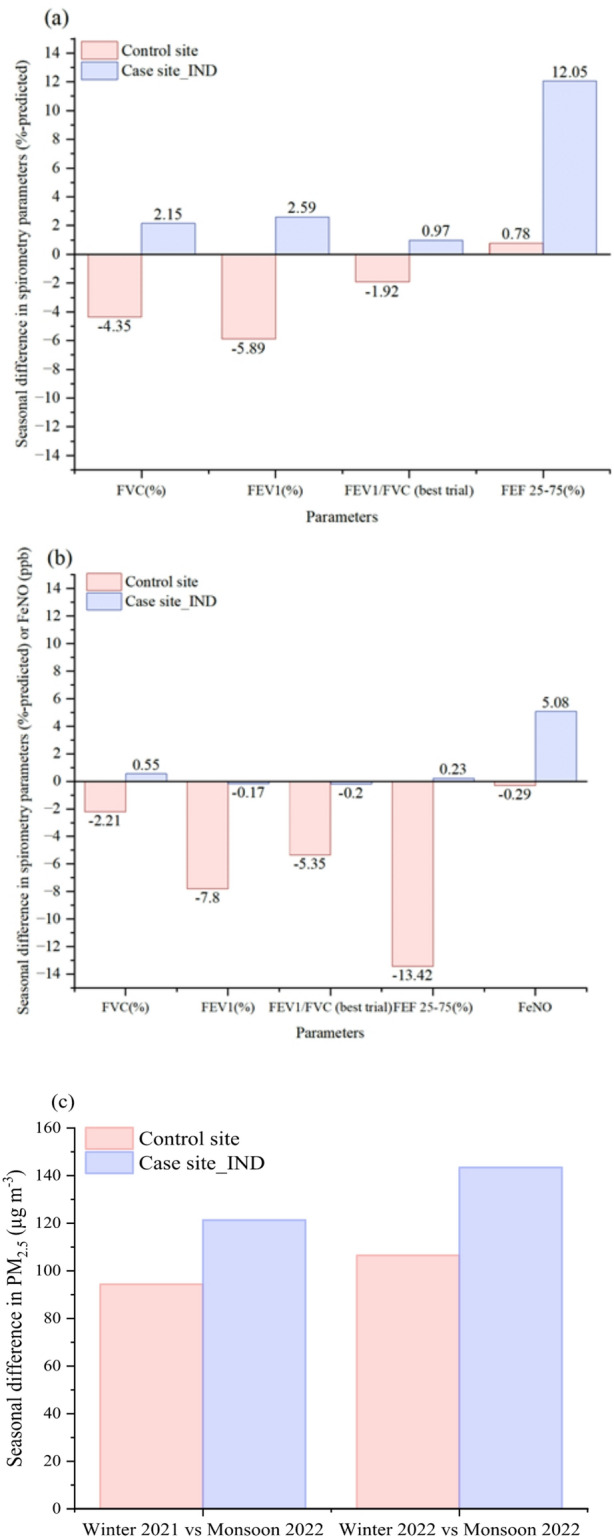
Seasonal differences (winter – monsoon) in spirometry parameters, FeNO values, and PM_2.5_ concentration at the case and control sites in India. Differences were calculated by subtracting the averaged monsoon 2022 values from winter 2021 for spirometry in panel (**a**) and winter 2022 for spirometry and FeNO in panel, (**b**) Similar measures for PM_2.5_ are presented in panel, (**c**) Parameters showing significant seasonal improvement at the control site include FEV_1_**, FEV_1_/FVC* in panel (**a**) and FEV_1_**, FEV_1_/FVC*** FEF_25-75_*** in panel (**b**). Parameters showing significant seasonal improvement at case site IND include FEF_25-75_* in panel (**a**) and FeNO in panel (**b**). *, **, *** represent statistically significant differences at 10%, 5%, and 1% levels of significance, respectively. The seasonal improvement in PM_2.5_ concentration is significant in all cases at the 5% significance level. Refer to Table S2 in Supplementary Information.

Seasonal differences in spirometry parameters, FeNO, and PM_2.5_ are calculated by subtracting the averaged values for monsoon 2022 from those for winter 2021 and 2022 (Fig. 4). The seasonal variation in ambient PM_2.5_ concentration, vis-à-vis spirometry results, suggests that even a sharp seasonal decline (by a factor of 3–4) in PM_2.5_ during the monsoon is not associated with any significant improvement in average respiratory health at the case site IND (Fig. 4). However, at the control site, most spirometry parameters show significant improvements (*p* ≤ 0.05), except for FEF_25−75_ and/or FVC, for which the seasonal changes are not statistically significant. The data in Table 2 clearly show that this disproportionate improvement in respiratory health results in considerable differences between the two sites across all spirometry parameters in monsoon 2022. 2022.

At the control site, FeNO levels do not change significantly, whereas at the case site IND, FeNO decreases moderately (*p* ≤ 0.09) during the monsoon compared with winter. All FeNO values, however, remain below 25 ppb. These findings support the hypothesis that, at the case site IND, the impact of pollution exposure on lung health is chronic and does not improve even when PM_2.5_ levels decline during the monsoon. However, for people with better or even normal lung function who live upwind of TPP, improved air quality translates into some improvement in lung function.

### The interplay of gender, tobacco, biomass use, and occupation

While the results indicate a potential association between exposure to TPP emissions and the respiratory health of subjects at downwind locations, several confounding factors, operating in complex interplay, likely influence the actual outcome. In this section, an effort is made to control for potential factors, such as gender, smoking, occupation, and exposure to emissions from solid fuel combustion during cooking^39–42^.

Population-based studies suggest that females are likely to have smaller absolute lung volumes than males, even when controlled for height^43^. With pooled data from the two sites in India, a similar trend is observed: females have lower FVC and FEV_1_ (in L) than males. The differences in winter 2021 FVC are statistically significant (*p* ≤ 0.01), with averages of 2.35 ± 0.56 L and 3.02 ± 0.77 L for females and males, respectively. Similarly, the average FEV_1_ is significantly lower (*p* ≤ 0.01) in females (1.9 ± 0.5 L) than in males (2.4 ± 0.7 L). Although the level of statistical significance is not maintained during the monsoon 2022 and winter 2022 measurement rounds, the pattern remains the same. However, the pattern is reversed for the predicted values (Table 3) that represent lung performance compared to healthy subjects with the same anthropometric parameters. Males exhibit significantly lower predicted values for FVC and FEV_1_ than females; these differences are statistically significant during monsoon 2022 and winter 2022 (*p* ≤ 0.01). Additionally, these values fall below the 80% threshold, suggesting an increased risk of obstructive pulmonary conditions and small-airway narrowing. While the ratio of FEV_1_/FVC is preserved (70–85%) in both males and females and both groups exhibit signs of PRISm, the values are significantly lower (*p* ≤ 0.01) in males than in females (Table 3).

We hypothesise that several factors may contribute to relatively poor respiratory health in males: outdoor occupational exposure, indoor biomass burning, and smoking. To account for smoking-related exposure, average spirometry values for males are recalculated after excluding smokers from both sites. None of the female participants in the study reported smoking, as it is considered a cultural taboo. Therefore, the difference between ‘all male and female’ and ‘all non-smoker male and female’ (Table 3) may capture the effect of smoking. The associated p-values suggest that the difference between ‘all male and female’ and ‘all non-smoker male and female’ is rarely significant. This finding is consistent with the relatively low smoking prevalence in the sample. It is also important to note that smoking status is self-reported, and underreporting by undisclosed smokers could have skewed the results.

After accounting for smoking, potential exposure can be attributed to two primary sources: (1) ambient air pollution, particularly for individuals in occupations requiring prolonged outdoor activity (e.g., farmers, drivers, and local vendors), and (2) indoor air pollution, especially for those involved in cooking with solid biomass fuels. To assess the effect of occupational exposure to ambient air pollution, we compared outdoor workers with others after excluding smokers and subjects exposed to biomass-based emissions from cooking. However, this analysis has limited statistical power, as the sample sizes were small (*n* = 9 at the control site and *n* = 5 at the case site IND). Despite this limitation, the results consistently indicated greater respiratory impairment at case IND, characterized by PRISm (FEV_1_/FVC ≈ 76%, with FEV_1_ and FVC < 80% predicted and FEF_25−75_ ≈ 65%), suggesting that prolonged outdoor exposure contributes disproportionately to deterioration of lung health among males at the downwind site.

The mean difference in the test comparing females who use biomass to those who do not (across both Indian sites) confirms that females who use biomass for cooking have poorer respiratory health (Table 4). However, the differences between these two groups are not statistically significant (except for FEV1/FVC in winter 2022 and FVC in the monsoon 2022 cycle), which may be due to the disproportionately small sample size of females who do not use biomass, especially in the winter 2022 cycle. During the monsoon, most spirometry parameters improve for both biomass users and non-users; however, the improvement is more pronounced among non-users. Consistent with this, a notable improvement in the average FVC is observed in females during the 2022 monsoon (Table 3). When this result is interpreted alongside the results in Table 4. It can be observed that the improvement in respiratory health (during the monsoon season) among females who do not use biomass contributes more to this increase. Although the statistical significance of the result is weak (*p* = 0.06), it nevertheless highlights a critical point: females who use biomass for cooking are more susceptible to deteriorating respiratory health, with limited scope for recovery, even during seasons typically associated with lower air pollution burdens.

**Table 3:** Differences in overall spirometry (percent predicted) across seasons for pooled Indian sites, with separate columns for all males, non-smoker males, and all females. Results indicate preserved FEV_1_/FVC with lower FVC and FEV_1_ in males, consistent with PRISm and elevated small-airway risk.

	Parameter	All males (*n* = 65)	All non-smoker males (*n* = 48)	All females (*n* = 72)	*p*-value for difference between males and females	*p*-value for difference between non-smoker males and females
Winter 2021	FVC (%)	79.4 ± 17.4	80.7 ± 17.2	81.2 ± 16.6		
	FEV_1_(%)	76.2 ± 19.1	78.0 ± 18.2	78.7 ± 16.4		
	FEV_1_/FVC – best trial	78.9 ± 9.8	79.9 ± 8.9	83.1 ± 8.4	0.00***	0.02**
	FEV_1_/FVC (%)	97.3 ± 11.2	98.3 ± 10.1	99.8 ± 10.1	0.08*	
	FEF 25–75(%)	72.1 ± 35.9	75.1 ± 37.9	69.4 ± 27.3		
	**Parameter**	**All male (n = 44)**	**All non-smoker males (n = 31)**	**All females (n = 43)**		
Monsoon 2022	FVC (%)	76.74 ± 17.7	75.5 ± 17.6	89.2 ± 21.0	0.00***	0.00***
	FEV_1_(%)	75.8 ± 21.5	74.4 ± 20.2	86.8 ± 21.0	0.00***	0.00***
	FEV_1_/FVC – best trial	81.0 ± 9.00	81.6 ± 8.0	83.9 ± 5.2	0.03**	0.08*
	FEV_1_/FVC (%)	103 ± 11.8	103 ± 10.4	102 ± 7.8		
	FEF 25–75(%)	64.8 ± 28.7	65.1 ± 26.4	69.3 ± 21.8		
	**Parameter**	**All male (n = 37)**	**All non-smoker males (n = 25)**	**All females (n = 57)**		
Winter 2022	FVC (%)	75.9 ± 17.3	75.6 ± 18.9	83.3 ± 11.8	0.01***	
	FEV_1_(%)	70.5 ± 19.4	71.2 ± 20.1	77.8 ± 13.5	0.02**	
	FEV_1_/FVC – best trial	76.2 ± 10.4	77.7 ± 9.5	80.4 ± 8.8	0.01***	
	FEV_1_/FVC (%)	96.8 ± 12.7	98.5 ± 11.6	98.1 ± 10.3		
	FEF 25–75(%)	53.6 ± 26.4	56.9 ± 27.9	59.9 ± 22.4		

*^,^**^,^***Represent statistically significant differences at 10%, 5%, and 1% levels of significance, respectively. The difference between the two categories ‘All male’ and ‘All non-smoker males’ are not statistically significant for any of the parameters and hence they are not separately mentioned in the table.

**Table 4:** Spirometry comparisons among females who use biomass vs. those who do not (pooled Indian sites), by season. Findings suggest a less pronounced seasonal recovery among biomass users and significantly lower FEV₁/FVC (winter 2022) and FVC (monsoon 2022) ratios.

	Variables	Females who do not use biomass (both sites combined; n = 11)	Females who use biomass (both sites combined; n = 39)	*p*-value for difference between the two groups
Winter 2021	FVC (%)	77.6 ± 16.5	82.7 ± 18.3	
	FEV_1_(%)	78.4 ± 16.5	78.6 ± 17.8	
	FEV_1_/FVC – best trial	85.9 ± 3.9	81.2 ± 9.4	0.01***
	FEV_1_/FVC (%)	104 ± 4.6	98.0 ± 11.4	0.00***
	FEF 25–75(%)	76.1 ± 19.3	67.0 ± 27.8	
		**Females who do not use biomass (both sites combined; n = 8)**	**Females who use biomass (both sites combined; n = 24)**	**p-value for difference between the two groups**
Monsoon 2022	FVC (%)	101 ± 18.9	87.5 ± 22.0	0.06*
	FEV_1_(%)	96.2 ± 16.9	85.8 ± 21.7	
	FEV_1_/FVC – best trial	83.0 ± 6.2	84.1 ± 3.9	
	FEV_1_/FVC (%)	101 ± 7.9	103 ± 6.2	
	FEF 25–75(%)	79.0 ± 96.2	67.0 ± 19.2	0.08*
	**Variables**	**Females who do not use biomass (both sites combined; n = 2)**	**Females who use biomass (both sites combined; n= 37)**	**p-value for difference between the two groups**
Winter 2022^#^	FVC (%)	79.6 ± 6.0	84.1 ± 12.6	
	FEV_1_(%)	77.4 ± 9.2	79.1 ± 14.9	
	FEV_1_/FVC – best trial	83.9 ± 2.9	80.2 ± 8.1	
	FEV_1_/FVC (%)	103 ± 6.4	98.7 ± 9.2	
	FEF 25–75(%)	71.5 ± 30.4	61.0 ± 24.6	

^*, **, ***^Represent statistically significant differences at 10%, 5%, and 1% levels of significance, respectively. ^#^results are not analyzed since there are only two observations under the first category.

The spirometry parameters are compared between (1) non-smoking males exposed to ambient air pollution who do not cook and (2) females (all non-smokers) who cook with solid biomass fuels but have limited ambient exposure due to their occupation. This comparison can shed light on how the average lung function of males primarily exposed to ambient air pollution differs from that of females primarily exposed to biomass-based indoor air pollution. There are 14 and 22 such subjects at the two Indian sites. Results suggest that all spirometry parameters are comparable for these two groups, indicating no significant difference in the effects of ambient and indoor air pollution on respiratory health. This exercise should be noted as having limited statistical relevance due to its small sample size.

## Discussion

**Fig. 5:**
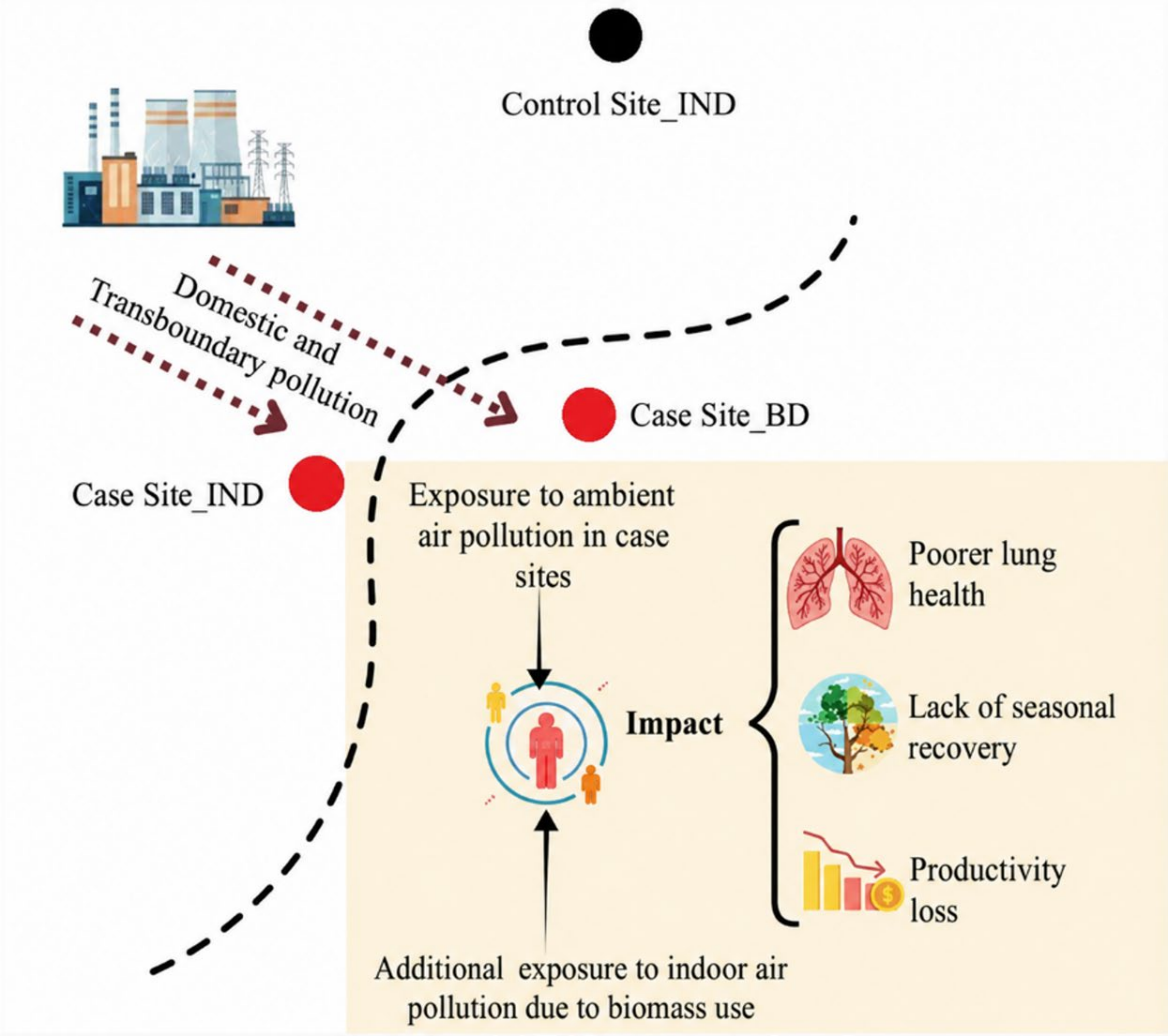
Visual summary of key findings: (i) higher downwind PM_2.5_ and poorer spirometry (PRISm, reduced FEF₂₅–₇₅), (ii) limited seasonal recovery at case IND despite monsoon PM_2.5_ declines, (iii) elevated wage/productivity loss, and (iv) amplification of risks by socioeconomic disadvantage and biomass use (“triple jeopardy”). IND and BD stand for India and Bangladesh sites in the summary figure.

Results suggest that the case sites are exposed to higher concentrations of ambient air pollution and are associated with poorer overall lung health than at the control site. Furthermore, signs of seasonal recovery in lung health are not apparent at the case site IND, even during the monsoon, and productivity loss is also higher. A visual summary of the results is presented in Fig. 5.

Benchmarking our spirometry observations against regional cohorts suggests substantial external validity. Data from the Indian subcontinent, where mean ambient PM_2.5_ levels are ~ 33 µg m^− 3^, report that higher PM_2.5_ exposure is associated with progressive FEV_1_ decline, indicating an obstructive/small-airway physiological signature^44^. This is consistent with Asian cohort data demonstrating chronic particulate exposure-related reductions in FEV_1_, FVC, and flow-dependent indices^45,46^, reinforcing the biological plausibility of pollution-mediated airway remodeling and impaired expiratory mechanics.

### Transboundary pollution

Trajectory modelling and ambient PM_2.5_ measurements indicate that communities in both India and Bangladesh are affected by local and regional pollution sources that extend beyond national borders. There is strong evidence that elevated PM_2.5_ exposure is linked to worsening respiratory health among populations downwind of the TPP, even in the presence of confounding factors. Overall, these findings underscore a critical transboundary dimension of the air pollution problem in this region, with implications that are not only environmental but also geopolitical. Careful site selection and strategic placement of large point sources are essential, especially near international borders and densely populated areas, to mitigate cross-border pollution and associated health risks. While proximity to coalfields, water resources, and labour markets remains important for operational efficiency, future energy planning must also integrate measures to minimize exposure and promote health equity. It is imperative that decisions regarding new point sources explicitly evaluate the vulnerability of surrounding populations and ecosystems, not just economic or logistical factors.

### Do socioeconomic conditions moderate health outcomes?

Globally, communities living downwind of coal-fired TPPs tend to have lower incomes and lower levels of education, which increases their susceptibility to pollution-related health risks^10^. This pattern is evident in the present study: the downwind site in India has much lower income and education levels than the upwind site and shows significantly greater deterioration in respiratory health. While this distribution may be partly coincidental, it nonetheless illustrates an important feedback loop: environmental exposure and socioeconomic disadvantage reinforce one another, creating a self-perpetuating cycle of vulnerability.

Globally, lower income levels in downwind locations have been linked to productivity losses from exposure to ambient PM_2.5_, as documented in the literature^41^. Our findings reinforce this pattern: residents at the downwind Indian site, already burdened by economic hardship, face additional productivity and wage losses due to declining lung function, further intensifying poverty. Notably, respiratory health deterioration is more pronounced among socioeconomically disadvantaged communities, even when PM_2.5_ exposure levels are comparable to those in upwind areas, suggesting that social vulnerability amplifies biological susceptibility. This interlinked cycle of poor health, reduced economic capacity, and continued exposure perpetuates vulnerability, forming a self-reinforcing loop that is difficult to disrupt without coordinated interventions in energy production, pollution mitigation, and socioeconomic support.

Indoor exposure from biomass burning further intensifies this risk. The results indicate that respiratory impairments among women who use solid biomass fuels are comparable to those from occupational exposure to ambient air pollution, confirming that indoor and ambient sources impose similar health burdens. Although males are more numerous in the second category, females dominate the first. These results, once again, emphasize the role of socioeconomic factors. Therefore, the risk to the exposed population is further elevated when biomass fuel use is widespread. In rural settings, financial constraints and limited education perpetuate reliance on solid biomass fuels despite their known health hazards^47^. Consequently, individuals at the Indian downwind site are often co-exposed to indoor and ambient pollutants, placing them among the most at-risk populations. This intersection of ambient exposure, poverty, and biomass dependence constitutes a “triple jeopardy” scenario in which environmental, economic, and social stressors interact to amplify health risks. The findings thus reinforce the need for targeted interventions, including access to cleaner energy, enhanced awareness, and localized health surveillance, to reduce vulnerability in these communities.

At the physiological level, average FEV_1_ values below 80% predicted, together with PRISm and FEF_25−75_ values below 65%, indicate that the downwind population is at substantial risk of developing obstructive pulmonary disease. PRISm, characterized by a preserved FEV_1_/FVC ratio (≥ 0.70) and reduced FEV_1_ (< 80% predicted), is an early yet often overlooked indicator of chronic airway pathology. Many individuals with PRISm later progress to clinically significant obstruction and increased mortality risk^48–50^. The argument for increased future vulnerability is further strengthened by lower FEF_25−75_ (< 65%) at the case site compared with the control site. Inflammation and narrowing typically begin in the small airways and then progress upward into the larger airways. A lower FEF_25−75_, a surrogate marker for small airway obstruction, indicates an early stage of disease before other spirometry parameters become abnormal. Notably, small airway inflammation and pathology precede the development of emphysema. Early identification and intervention in such cases could help mitigate progression to irreversible disease.

### Is the damage reversible?

Numerous epidemiological studies have shown that even brief exposures (daily to weekly) to PM_2.5_ can diminish lung function, but these short-term effects may be reversible. In contrast, prolonged exposure leads to significantly more pronounced effects and is associated with reduced lung function that may be irreversible^51,52^. Spirometry parameters indicate greater narrowing of the airways at the Indian case site than at the control site, and the impairment is more likely to be ‘permanent’ than temporary. The narrowing of the airways could be due to physical constriction or inflammation of the mucous lining. While physical narrowing associated with smooth muscle thickening is irreversible, inflammation, i.e., swelling and redness that reduce the airway diameter, can be reversed. Inflammation of the airways is usually associated with elevated FeNO levels. Therefore, lower FEV_1_ values with non-elevated FeNO levels at the case site indicate that the change in lung physiology of the exposed population is likely to be permanent and less likely to be reversed. This is also corroborated by the fact that, while a significant improvement in lung function is observed at the control site due to a sharp decline in PM_2.5_ levels during the monsoon, the improvement is negligible at the case site.

## Conclusions and limitations

The primary question this work seeks to address is whether ambient air pollution from a coal-fired TPP near the India-Bangladesh border is significantly associated with the respiratory health of the exposed population on a transnational scale. Although the answer is far from straightforward because of confounding factors, this study provides evidence that communities at the downwind site near the India–Bangladesh border experience impaired respiratory health across national boundaries, with patterns consistent with the chronic effects of prolonged exposure to PM_2.5_. The findings reveal higher rates of reduced lung function, PRISm, and small-airway obstruction at downwind locations, accompanied by productivity and wage losses. Importantly, lung function did not recover during the monsoon season, when air quality is better due to lower PM_2.5_ levels, indicating chronic rather than seasonal effects. Socioeconomic disadvantages and widespread biomass fuel use further magnify the risk, creating a triple burden of exposure, poverty, and limited adaptive capacity. In a country like India, where average PM_2.5_ levels routinely exceed WHO standards, these results underscore the urgent need to transition to cleaner energy sources for power generation and household use, and to locate industrial facilities responsibly away from densely populated areas.

Finally, we highlight potential limitations of the current study. First, data collection was limited to three seasons in India and one in Bangladesh. Including additional time points in this longitudinal study would have helped distinguish between short-term and chronic health effects. Second, real-time indoor measurements of PM_2.5_ and/or CO levels would have been beneficial, enabling better characterization of indoor exposure. Third, the study does not distinguish between pre-existing and newly developed respiratory conditions. Future research should extend this longitudinal framework by incorporating additional temporal sampling, integrating indoor–outdoor air-quality monitoring, and employing advanced statistical modeling (e.g., mixed-effects and structural equation models) to disentangle causal pathways and explore adaptive or mitigating interventions. Fourth, we acknowledge that observed socioeconomic differences between sites, particularly the lower income and education levels at the case site IND, raise the possibility of residential sorting. This suggests that households with fewer resources may preferentially locate in downwind areas, which may have lower land values. While simple random sampling and the breadth of the household survey allow a partial examination of this issue, future work should employ quasi-experimental or propensity-score matching designs to more rigorously isolate the effect of TPP emissions on pre-existing socioeconomic disadvantages.

## Supplementary Information

Below is the link to the electronic supplementary material.

Supplementary Material 1

## Data Availability

The analysis is conducted based on an extensive primary dataset. Data will be made available on request.
