# Supplementary material for: Respiratory health impacts of coal-fired power plant emissions with transboundary environmental complexities
Source: Sci Rep. 2026 Jun 29;16:20544. doi: 10.1038/s41598-026-58245-z (PMC13332190; doi:10.1038/s41598-026-58245-z)
Supplement: Supplementary file 1 — Supplementary Material 1 [file 41598_2026_58245_MOESM1_ESM.docx]

# **Supplementary material**

**Section S1. Details of instruments and statistical analysis**

**Air sampler**: At the Indian sites, high-volume air samplers (Hi-Vol 3000, Ecotech) with flow rates of 1.2 m^3^ min^-1^ were used to collect PM_2.5_ on a 24-h basis on pre-combusted (550°C for 5 h) 8” x 10” quartz microfiber filters (QMA, Whatman). At the Bangladesh site, a low-volume sampler (APM 550MFC, Envirotech) with a flow rate of 16.7 L min^-1^ was used to collect PM_2.5_ on 47 mm diameter quartz microfiber filters (QMA, Whatman). PM_2.5_ loadings were determined gravimetrically using a microbalance (Mettler-Toldeo, sensitivity: 0.01 mg) after conditioning in a desiccator at constant temperature and relative humidity for 48 h before and after sampling. Filters were stored at −4°C. While this work only refers to PM_2.5_ mass concentrations, a comprehensive suite of speciation measurements has been conducted on these samples, including organic and elemental carbon, water-soluble organic carbon, ionic species, major, trace and rare earth elements, polycyclic aromatic hydrocarbons, n-alkanes, levoglucosan, and dithiothreitol-based oxidative potential and •OH generation. These results will be reported elsewhere.

**Spirometry:** Spirometry was performed using an auto-calibrated PC-driven spirometer (Easy on-PC, Medical Technologies, USA) on subjects in a sitting posture as per American Thoracic Society (ATS)/European Respiratory Society (ERS) recommendations. Reproducibility of results, i.e., FEV1 and FVC, each varying by ≤150 mL between corresponding largest and next-largest values, was ensured when selecting acceptable tests.

**FeNO:** FeNO analysis was conducted as per ATS/ERS guidelines at a fixed exhalation flow rate of 50 mL s^-1^ using NIOX VERO (NIOX Group, UK) that employs an electrochemical sensor with in-built zero gas measurement. The instrument has a measurement range of 5-300 ppb with precision and accuracy of ≤10% at ≥50 ppb. Agreement within 10% of at least two NO measurements was considered to be reproducible.

**Statistical Analysis**: Descriptive statistics were initially generated to elucidate the average values of socioeconomic indicators, PM_2.5_ levels, spirometry, and FeNO results. Mean values of respiratory health parameters were statistically compared between the case and control sites and across seasons using *t*-tests to determine any significant difference. Variances were tested for equality before carrying out a *t*-test for mean values. In observational studies like this one, where subjects interact with their natural environment without control over external variables, it is essential to consider confounding factors. These factors are external variables that may influence the study outcome independently of the investigated variable. Therefore, we compared lung function parameters to draw accurate conclusions after controlling for gender, occupation, tobacco exposure, and biomass fuel use at the case and control sites. By doing so, the study aims to isolate better the impact of the primary variable (in this case, the effect of TPP emissions on respiratory health) while minimizing bias from unrelated influences. Further, kernel density plots were generated for the standardised values of wage loss and productivity (workday lost). Kernel density plots represent an approximation of the probability density function of the variable under consideration. The wage loss and productivity loss are calculated, respectively, by adding the reported wage and the workdays lost by the patient and the caregiver (if any). The observations are standardised by dividing the deviation from the mean by the standard deviation. The statistical software STATA 16.1 was used to carry out the analyses.

**Table S1. Summary of interpretation of spirometry parameters and FeNO**

| **Lung health parameters** | **Interpretation and use** | **Range of %-pred*** | **Remarks** |
| --- | --- | --- | --- |
| **Spirometry** | | | |
| FVC: forced vital capacity | The amount of air a person can forcibly exhale after the deepest possible breath. Lower values indicate restricted breathing potentially related to restrictive airway disease (reduction in the size of the lungs), lung inflammation, lung cancer, etc. | 80-120% | Normal |
|  |  | <80% | Indicative of restricted breathing |
| FEV_1_: forced expiratory volume in 1 second | The maximum amount of air a person can exhale after the deepest possible breath. The lower the FEV1 value, the greater the severity of obstructive lung function impairment in the lower airways. | 70 - 79% | Mild |
|  |  | 60 - 69% | Moderate |
|  |  | 50 - 59% | Moderately severe |
|  |  | 35 - 49% | Severe |
|  |  | <35% | Very severe |
| FEV_1_/FVC ratio | Ratio of FEV1 and FVC. A lower value indicates chronic airway obstruction, i.e., narrowing of the airways in the lungs due to physical obstruction or dynamic airway collapse.  Note:   1. Lung restriction reduces FEV_1_, FVC, but not the FEV_1_/FVC ratio. 2. Low value of FVC with increased FEV_1_/FVC to 85–90% is indicative of restrictive lung function impairment. 3. Preserved FEV_1_/FVC ratio along with lower levels of FVC or FEV_1_ is characterised as preserved ratio impaired spirometry (PRISm), indicative of early COPD. | <70% | Indicative of obstructive diseases |
| FEF25-75%^#^: forced expiratory flow between 25% and 75% of vital capacity | The average flow is between 25% and 75% of exhaled FVC. A lower FEF25-75% value is a surrogate marker for obstructive peripheral airflow and is indicative of early bronchial impairment associated with small airway disease. | <65% | Indicative of obstructive disease |
| **FeNO** | | | |
| FeNO: fractional exhaled nitric oxide | Measures the amount of nitric oxide (NO; in ppb) exhaled from a breath. An elevated NO level indicates airway inflammation. | <25ppb | Absence of airway inflammation |
|  |  | 25-50ppb | Controlled airway inflammation |
|  |  | >50ppb | Uncontrolled airway inflammation |

*All spirometry values other than the FEV_1_/FVC ratio mentioned here are in terms of %-predicted (=Observed*100/Predicted), i.e., compared to the average for someone of the same age, height, sex, and ethnicity. In the analysis, for FEV_1_/FVC both %-predicted and best trial values are interpreted. ^#^ Note that FEF25–75 is not recommended by the ERS/ATS guidelines on interpretation of spirometry for the diagnosis of asthma. The ranges and interpretations provided here are based on Stanojevic et al. (2022), Agusti et al. (2023), Kwon et al. (2020) and Dweik et al. (2011).

**Table S2. Seasonal differences in spirometry parameters and FeNO values at the case and control sites in India (data and statistical test supporting Figure 4)**

|  | **Variables** | **Malda** | **p-value Malda** | **Murshidabad** | **p-value Murshidabad** |  |
| --- | --- | --- | --- | --- | --- | --- |
| **Difference between Winter 2021 and Monsoon 2022** | **FVC(%)** | -4.35 | 0.1 | 2.15 | 0.28 |  |
|  | **FEV1(%)** | -5.89 | 0.04** | 2.59 | 0.26 |  |
|  | **FEV1/FVC (Best Trial)** | -1.92 | **0.06*** | 0.97 | 0.32 |  |
|  | **FEF 25-75(%)** | 0.78 | 0.43 | 12.05 | 0.02** |  |
|  | **FeNO** | - |  | - |  |  |
| **Difference between Winter 2022 and Monsoon 2022** | **FVC(%)** | -2.21 | 0.27 | 0.55 | 0.44 |  |
|  | **FEV1(%)** | -7.8 | 0.01** | -0.17 | 0.48 |  |
|  | **FEV1/FVC (Best Trial)** | -5.35 | **0.00***** | -0.2 | 0.46 |  |
|  | **FEF 25-75(%)** | -13.42 | 0.00*** | 0.23 | 0.48 |  |
|  | **FeNO** | -0.29 | 0.45 | 5.08 | 0.09* |  |

Differences were calculated by subtracting the averaged monsoon 2022 values from the winter 2021 and winter 2022 values. *, **, *** represent statistically significant differences at 10%, 5%, and 1% levels of significance, respectively.

**References**

Augusti, A., Celli, B.R., Criner, G.J., Halpin, D., Anzueto, A., Barnes, P., et al., 2023. Global initiative for chronic obstructive lung disease 2023 report: GOLD executive summary. Eur. Respir. J. 61, 2300239.

Dweik, R.A., Boggs, P.B., Erzurum, S.C., Irvin, C.G., Leigh, M.W., Lundberg, J.O., Olin, A-C., Plummer, A.L., Robin Taylor, D., 2011. An official ATS clinical practice guideline: interpretation of exhaled nitric oxide levels (FeNO) for clinical applications. Am. J. Respir. Crit. Care Med. 184, 602-615.

Kyon, D.S., Choi, Y.J., Kim, T.H., Byun, M.K., Cho, J.H., Kim, H.J., Park, H.J., 2020. FEF25-75% values in patients with normal lung function can predict the development of chronic obstructive pulmonary disease. Int. J. Chron. Obstruct. Pulmon. Dis. 15, 2913-2921.

Stanojevic, S., Kaminsky, D.A., Miller, M., Thompson, B., Aliverti, A., Barjaktarevic, I., et al., 2022. ERS/ATS technical standard on interpretive strategies for routine lung function tests. Eur. Respir. J. 60, 2101499.
